# Better Training for Matrons

**Published:** 1909-02-06

**Authors:** 


### NURSING ADMINISTRATION.

### BETTER TRAINING FOR MATRONS.

### V.—THE PRACTICE OF HOUSEKEEPING.

HOUSEKEEPING in the ordinary sense of the word is the most unscientific pursuit to which the energies of woman can be directed. It is not too much to say that, conducted under advanced principles of efficiency and economy, it is sufficient to tax the powers of the ablest administrator. It is work which can only be learned under the personal direction of a good chief, by actually performing the duties of which it is composed. Books are useful, but to study housekeeping through the medium of books never leads to efficiency. Schools for housekeepers, warmly advocated in some quarters, are better than no instruction at all, but it would be as futile to expect a woman to learn nursing in an establishment devoid of patients, as to expect her to become a good housekeeper in an establishment with only students for inmates. The only proper conditions under which institution housekeepers can be trained are to be found within the walls of the institution, under the direction of an able housekeeper, whose duties include the training of subordinates. It is too often the case that all the valuable material for teaching purposes presented by the housekeeper's department in institutions is wasted from some parsimonious idea that to put an extra hand to work under the housekeeper is unnecessary. Very often the matron is deprived of any assistance in the matter of housekeeping, except such as she can spare in seasons of least pressure from some other department. And the direct consequence of this is that the housekeeping is and must be neglected, for it is a business which will not endure to take the second place. Our experience goes to show that there is hardly a hospital in the kingdom where the presence of a third or fourth year student probationer in the housekeeping department for six months at a time would not be a source of strength to the establishment generally, setting the matron or housekeeper free for more careful supervision, lessening the rush which makes the wheels move irregularly, and last, but not least, aiding to perfect the system. The parallel between nursing and housekeeping again holds good, for just as no full current of life and perfection of organisation can exist in a hospital where no training of probationers is carried on, even so a housekeeping department in which no one is keenly interested either to learn or to teach speedily grows slipshod and loses the first rank. In institutions of great size, where several assistant matrons are employed, it would be possible to place a student under each, so that they could pass on in regular rotation as soon as they had mastered the routine in each department. But the routine is the least part of the business which would be imparted under a live system of training. The daily mechanism must of course be mastered as part of the work of a good housekeeper, but the student would be introduced to the springs which underlie the routine. She would study, for instance, when engaged in the commissariat department, the various contracts, and even where these matters are in the steward's department, would have opportunity given her to understand distinctions in price and quality. She would be expected to master the great law of comparisons which rules economy in big establishments, and in discovering the difference which blue instead of green plums can make in the quarter's average she will be on the road to victual her own household when she has one, with distinction. There would be very little extra trouble involved in teaching the secrets of housekeeping to a succession of keen and already trained women. It would mean that those on whose shoulders the teaching rested would be compelled to be sure of their own reasons for particular methods, but whatever time was consumed in explanations would be saved a thousand fold by the assistance of an intelligent coadjutor. Through the whole course would run the bracing influence of an examination in the future, and teacher and pupil alike would be guided by a curriculum sketched out by competent authorities. Such a housekeeping course as would fit a woman for the discharge of her duties in the administration of an institution, whether large or small, would include the purchase of provisions by contract or selection, their price and quality, including the study of English and foreign meat, groceries, butter, bread, milk, etc.; the superintendence of the kitchen, and the management of the more ordinary utensils and cooking apparatus in use in large establishments; the control of gas and electric light; the price and quality of coal suitable for use in institutions; the best methods of cleansing floors, etc., of controlling the use of material for cleaning purposes, and of supervising the workers; a thorough knowledge of the wardmaids' and housemaids' work, with an insight into the system of engaging domestics, and of training them in the performance of their work. These are but some of the more obvious directions in which an examiner would expect the student for the higher certificate of hospital administration to show herself proficient. It is certain that the inauguration of such examinations would call into being handbooks on all these subjects, now unhappily studied in but few establishments with true enthusiasm. How long would suffice for the course we have lightly sketched? It can hardly be claimed that a two years' course would give proficiency in every department, but it would undoubtedly give such an insight into the matter as could be perfected in subsequent posts. The material lies ready to hand in a few well-administered institutions. It remains only to make it of the utmost value to the ceaseless procession of intelligent women who are now allowed to pass in and out of these walls without ever detecting the secret which makes the machine revolve tranquilly in its diurnal motion.

